# Very low doses of rituximab in autoimmune hemolytic anemia—an open-label, phase II pilot trial

**DOI:** 10.3389/fmed.2024.1481333

**Published:** 2024-12-20

**Authors:** Miriam M. Moser, Renate Thalhammer, Christian Sillaber, Ulla Derhaschnig, Christa Firbas, Ulrich Jäger, Bernd Jilma, Christian Schoergenhofer

**Affiliations:** ^1^Department of Medicine I, Division for Infectious Diseases and Tropical Medicine, Medical University of Vienna, Vienna, Austria; ^2^Department of Laboratory Medicine, Medical University of Vienna, Vienna, Austria; ^3^Department of Medicine I, Division of Hematology, Medical University of Vienna, Vienna, Austria; ^4^Department of Clinical Pharmacology, Medical University of Vienna, Vienna, Austria

**Keywords:** rituximab, CD20+ B cells, autoimmune hemolytic anemia (AIHA), cold agglutinin disease, warm autoimmune hemolytic anemia

## Abstract

**Introduction:**

Although rituximab is approved for several autoimmune diseases, no formal dose finding studies have been conducted. The amount of CD20+ cells differs significantly between autoimmune diseases and B-cell malignancies. Hence, dose requirements of anti-CD20 therapies may differ accordingly.

**Methods:**

We conducted a phase II pilot trial investigating the effects and safety of very low doses of rituximab, i.e., 5 mg/m^2^ every 3 weeks, 20 mg every 4 weeks, 50 mg every 3 months (*n* = 3 each) and 100 mg every 3 months (*n* = 1) in patients with autoimmune hemolytic anemia (AIHA) to effectively suppress CD20^+^ cell counts. Doses were increased if circulating CD20^+^ cell depletion was insufficient (i.e., <95% reduction from baseline) in a dose group. Plasma rituximab concentrations were quantified by enzyme-linked immunosorbent assay, CD20^+^ cell counts were determined by flow cytometry.

**Results:**

Ten patients were included in the final analysis (7 with cold agglutinin disease, 2 with warm AIHA, 1 with mixed-type AIHA). The first infusion depleted ≥95% of CD20^+^ cells in all but one of the included patients. However, the dosing regimens were found ineffective, because a sustained CD20^+^ cell depletion was not achieved, and CD20^+^ cells recovered with a high interindividual variability. CD20^+^ lymphocytes were below the detection limit if rituximab plasma concentrations exceeded 0.4 μg/mL. One endokarditis occured.

**Conclusion:**

Rituximab doses as low as 5 mg/m^2^ transiently depleted CD20^+^ cells in almost all patients, but the tested low-dose regimens failed to permanently suppress CD20^+^ cells. The empirically identified EC95% of 0.4 μg/mL rituximab may guide future studies using low-doses of rituximab.

**Clinical trial registration:**

https://clinicaltrials.gov/, identifier [EudraCT 2016-002478-11].

## 1 Introduction

Rituximab, a chimeric monoclonal antibody, is directed against the CD20 antigen expressed on B cells and most of their precursors. It was the first ever approved therapeutic antibody and its initial label was the treatment of Non-Hodgkin Lymphoma with a recommended dose of 375 mg/m^2^ once weekly for 4 weeks (1). Meanwhile, it has become standard of care (mostly as part of an immune-chemotherapy) in various B-cell malignancies, such as diffuse large B-cell lymphoma, chronic lymphocytic leukemia, follicular lymphoma, and mantle cell lymphoma (2). Rituximab depletes CD20^+^ cells by either triggering complement-dependent cytotoxicity or initiating antibody-dependent cellular cytotoxicity (3–5). Beside its therapeutic effects in B-cell malignancies, rituximab also produces immunomodulatory effects, which led to its application in various autoimmune diseases (6–10). In rheumatoid arthritis the approved dose is 1,000 mg on day 1 and day 15 (7), while in ANCA-vasculitis 375 mg/m^2^ weekly for 4 weeks or 1,000 mg on day 1 and 15 are recommended (6). Rituximab is also approved in pemphigus vulgaris, when clinical remission is not achieved with systemic corticosteroids or other immunosuppressive drugs (rituximab 2 × 1,000 mg 2 weeks apart or 375 mg/m^2^ weekly x4) (8–10). Moreover, rituximab is frequently used “off-label” in other autoimmune diseases such as autoimmune hemolytic anemia (AIHA) (11), thrombotic thrombocytopenic purpura (12), immune thrombocytopenia (13), or multiple sclerosis, typically with four doses of 375 mg/m^2^ per week (14).

Although, the approval for rituximab dates back decades (1) and the pharmacokinetics (PK) of rituximab have been analyzed in numerous studies, only a single dose finding study was performed in 15 patients with relapsed low-grade B cell lymphoma (15, 16). However, there are no formal dose-finding studies for autoimmune diseases (15–26). This is especially interesting because B-cell malignancies are associated with a much higher antigen burden compared with autoimmune diseases, which may necessitate much higher and more frequent dosing in malignant diseases (18).

Several clinical studies already investigated alternative dosing regimens (like 200 mg, 250 mg/m^2^ or 500 mg) and overall showed comparable efficacy to standard doses, for instance, in cryoglobulinemia vasculitis or rheumatoid arthritis (27, 28).

In AIHA, autoantibodies directed against erythrocyte surface molecules lead to the destruction of erythrocytes and consecutively to anemia. According to the First International Consensus Meeting, corticosteroids remain first line therapy in AIHA adults (29), but targeting CD20^+^ cells by adding rituximab might contribute to a decrease in erythrocyte antibody production and therefore is recommended in severe cases (29, 30).

In former studies (31, 32) patients with warm AIHA or with cold agglutinin disease (CAD) received a fixed dose of 100 mg rituximab once a week over 4 weeks with response rates of about 90%. However, despite these studies proving efficacy of lower doses, the investigated dosing regimens were neither based on traditional dose finding studies nor derived from pharmacological parameters.

### 1.1 Hypothesis

In an earlier trial that included healthy volunteers, the half-maximal effective dose (ED50) of rituximab was approximately 0.1–0.3 mg/m^2^ (33). The half-maximal effective dose is defined as the dose at which a drug reaches 50% of its effect. This parameter is tightly connected to the EC50, which describes the concentration at which 50% of the response of a drug is achieved. These parameters relate to the steep part of the dose–response curve and are used to quantify and/or compare the potency of drugs, but may also be used to describe interindividual variability (34). At a dose of 1 mg/m^2^ rituximab reduced CD20^+^ cell counts transiently by 97% (the approximate ED95) (33). Based on these data, we estimated the EC95 to be approximately 600–700 ng/mL. The ED95 and the EC95 correspond to the dose and concentration at which a drug achieves 95% of its intended effect. These parameters, alongside other pharmacological parameters such as absorption or elimination, are important for dose finding and to develop therapeutic regimens. In an *in vitro* study the half-maximal effective concentration (EC50) of rituximab for depletion of human B cells was found to be approximately 1 μg/mL (35).

In sum, approved doses exceed these concentrations several hundred-fold supporting the hypothesis that much lower doses may be equally effective (details in Supplementary Figure S1) (15).

In this study we set out to investigate several dosing regimens that were calculated using these parameters in addition to the well-known terminal elimination half-life of rituximab of approximately 21 days in autoimmune diseases (36) and tested their effects in patients with AIHA. We hypothesized that in non-malignant diseases a long-lasting and effective suppression of CD20^+^ cells may be possible using much lower doses of rituximab.

## 2 Materials and methods

### 2.1 Study design

This was a phase II, open label, pilot trial to investigate the effects and the safety of very low doses of rituximab in patients with AIHA. We included patients ≥18 years with a diagnosis of AIHA in whom the treating physicians decided to use rituximab. Patients were excluded if they had received rituximab or other CD20-targeting therapies (e.g., ofatumumab, obinutuzumab or ocrelizumab) within the last 12 months and if they had suppressed (non-detectable) CD20^+^ cell counts at screening. Moreover, patients treated with high doses of corticosteroids (> 100 mg/day), or intravenous immunoglobulins, because of autoimmune-mediated hemolysis, were not eligible. Any concomitant medication was allowed. A detailed list of all in-and exclusion criteria is presented in Supplementary material. The study was designed to determine whether the different dosing regimens may suppress CD20^+^ cells for the projected treatment period. Depending on the dose group, the active treatment period lasted between 3 months and up to 9 months: four infusions every 3 weeks in the 5 mg/m^2^ group, three infusions every 4 weeks in the 20 mg fixed dose group, three infusions every 3 months in the 50 mg and 100 mg dose group. The duration of the active treatment phase was based on observations how long CD20^+^ cells are fully suppressed after administration of approved doses in autoimmune diseases (37, 38). The active treatment phase of the low dose groups (approximately 3 months) was deemed long enough to assess efficacy, but somewhat limited by the number of visits patients had to pay to the study ward. Of note, the study also included the option to continue treatment for up to 2 years (100 mg rituximab every 3 months), if patients clearly benefited from rituximab treatment.

The study was conducted at the Department of Clinical Pharmacology, Medical University of Vienna, between 2016 and 2021. All study procedures complied with the principles set forth in the Declaration of Helsinki and the Good Clinical Practics guideline. The Ethics Committee of the Medical University of Vienna approved the study before its initiation (EK 1630/2016, EudraCT 2016–002478-11). The COVID-19 pandemic had a significant impact on patient recruitment, because (i) there was a strategy to reduce patient contacts within the healthcare system and (ii) physicians were temporarily reluctant to use rituximab given an assumed negative impact on patient outcomes, which was later confirmed (39).

### 2.2 Study procedures

At the screening visit all patients underwent a physical examination, an electrocardiogram, a measurement of oral temperature and vital signs, blood and urine collection for clinical laboratory assays. On study days, vital parameters were monitored (heart rate, blood pressure and oxygen saturation) and recorded. A baseline blood sample was drawn including laboratory parameters indicative of hemolysis (haptoglobin, free hemoglobin, LDH, bilirubin, reticulocytes). All patients received premedication of 5 mg levocetirizine perorally, 1,000 mg paracetamol perorally, and 1,000 mL of 0.9% saline solution intravenously at least 30 min before the first rituximab infusion. Premedication at later time points could be omitted, if CD20^+^ cells were fully suppressed. The respective rituximab dose was diluted to a final volume of 20 mL and infused over 1 h. Blood sampling was performed 2 h after the end of the infusion and vital signs were measured on an hourly basis. The first control visits were conducted 1 and approximately 7 days after infusion and included the collection of adverse events, concomitant medication, as well as blood sampling and the measurement of vital signs. Based on CD20^+^ cell counts additional control visits could have been performed. Subsequent rituximab infusions (number 2–3 or 4, as applicable) were conducted according to the respective dosing regimen and with similar study procedures. After the active treatment phase control visits were performed approximately every 3 weeks to measure drug concentrations and CD20^+^ cell recovery. The final examination was performed 2–4 weeks after the last control visit and included blood sampling, measurement of vital signs, a physical examination, and an ECG.

### 2.3 Dosing rationale and regimen

In a previous clinical trial, healthy volunteers received very low doses of rituximab (0.1, 0.3, and 1 mg/m^2^) (33). Infusion of 0.1 mg/m^2^ and 0.3 mg/m^2^ reduced CD20^+^ cells transiently by 68 and 74% and infusion of 1 mg/m^2^ rituximab depleted CD20^+^ cells almost completely (~97%). Four weeks after the infusion of 1 mg/m^2^ rituximab CD20^+^ cell counts recovered to approximately 60% (33). The quantification of rituximab plasma concentration was somewhat complicated by a limited assay sensitivity and target mediated drug disposition that may be especially pronounced for very low doses. We calculated a basic PK model with the following assumptions: theoretical circulating blood volume of 3,000 mL, EC95 of ~600 ng/mL, ED95 of 1 mg/m^2^, ED50 of 0.1 mg/m^2^, and a terminal elimination half-life of ~21 days (36). The exact model is available as a Supplementary Figure S1. In short, we expected an infusion of 5 mg/m^2^ every 3 weeks (dose group 1), an infusion of 20 mg fixed dose every 4 weeks (dose group 2), an infusion of 50 mg fixed dose every 3 months (dose group 3) to effectively suppress CD20^+^ cell counts for the entire scheduled period. As a fourth dose group 100 mg every 3 months was amended to the protocol.

Doses were escalated, if the suppression of CD20^+^ cells was not sufficient in all patients of a cohort (defined as a 95% reduction of CD20^+^ cell counts from baseline).

### 2.4 Endpoints

The primary efficacy endpoint was an ≥95% suppression of CD20^+^ cells during the active treatment phase. Plasma concentrations of rituximab were quantified and pharmacodynamic endpoints included hemoglobin concentrations, and signs of hemolysis. Due to sparse blood sampling a full analysis of pharmacokinetics was not performed. However, we analyzed the pharmacokinetic/pharmacodynamic relationship in an exploratory manner.

Plasma rituximab concentrations were quantified by enzyme-linked immunosorbent assay (VelaLabs GmbH, Vienna, Austria). CD20^+^ cell counts were determined by flow cytometric analysis (Department of Laboratory Medicine, Medical University of Vienna, Austria).

Hemolysis-specific laboratory parameters included the following: hemoglobin concentration (local reference range 12–16 g/dL for females, 13.5–18 g/dL for male), reticulocyte counts (32–110 G/L), LDH (< 250 U/L), total bilirubin concentration (0.0–1.2 mg/dL) and haptoglobin concentration (30–200 mg/dL). Safety endpoints included vital signs, adverse events, and safety laboratory parameters.

### 2.5 Statistical analysis

Demographics and baseline data are presented by descriptive statistics (mean, standard deviation). The primary endpoint was a continuous ≥95% suppression of CD20^+^ cells in between dosing intervals. An exact confidence interval with a one-sided nominal coverage probability of 90% for the true rate of responders was calculated. Endpoints are presented descriptively.

In the forementioned trial (33), 16 healthy volunteers were enrolled. Subjects received 0.1 mg/m^2^ (4 subjects), 0.3 mg/m^2^ (4 subjects) or 1 mg/m^2^ (8 subjects) in a dose escalating manner. CD20^+^ cell counts showed a large variability with coefficients of variation of approximately 20–60%. The initial depletion of circulating B-lymphocytes was ≥95% after 1 mg/m^2^ rituximab infusion (33). Based on these results, strong treatment effects were expected in patients. For CD20^+^ cell numbers below the lower limit of quantification we imputed a CD20^+^ cell count of “0” and assumed treatment success. Treatment success was defined as a ≥95% reduction of CD20^+^ cells during the entire scheduled period. We desired a 100% response rate, which would result in the lower bound of a one-sided 90% confidence interval of 0.56, 0.68, 0.75 for the sample sizes *n* = 4, *n* = 6 or *n* = 8, respectively, to achieve sufficient evidence for the efficacy of very low-dose rituximab. In case a dose level was found inefficacious, the dose was escalated. We aimed for a minimum sample size of *n* = 3 per dose group. If the dose was inefficacious, we escalated the dose. However, if a dose effectively reduced CD20^+^ cell counts according to the defined criteria, more patients were to be enrolled in this dosing cohort. However, due to the early termination of the trial and, because the tested rituximab regimens were found inefficacious, the statistical analysis was done in a descriptive manner only.

Figures were illustrated with graphpad prism 10. MS excel, and SPSS were used for statistical calculations.

## 3 Results

**Figure 1:**
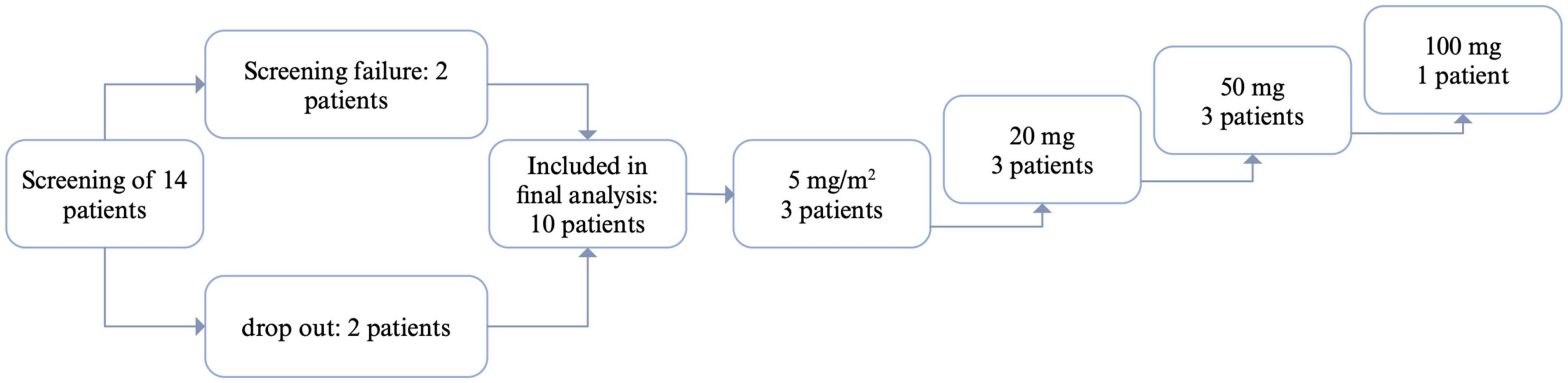
Flow-chart patient inclusion. CR, complete response: CD20+ cell count suppressed below 10% of baseline; PR, partial response: CD20 cell count suppressed below 10% of baseline, but increase in CD20 in between; NR, no response. Created with biorender.

A total of 14 patients were screened between 2016 and 2021. There were two screening failures (suppressed CD20^+^ cells in one patient, treatment with immunoglobulins in another patient). Ten patients completed the study, while two patients dropped out (Figure 1). Two patients suffered from warm AIHA, while seven patients suffered from cold agglutinin’s disease and one patient suffered from a mixed-type AIHA with autoantibodies compatible with cold agglutinin’s disease and warm AIHA. The study was terminated early due to slow recruitment of patients, especially during the COVID-19 pandemic.

The mean age was 68 years (±10 standard deviation) and the mean weight was 71 kg (± 13). Six female and four male patients completed the study. One patient received rituximab previously for treatment of autoimmune hemolytic anemia more than 12 months ago (Table 1).

**Table 1:** Patient demographics and baseline data.

Included patients		10
Mean age (years)		69 ± 10
Sex		
female		7
male		3
Type of autoimmune hemolytic anemia (AIHA)		
warm AIHA		2
cold agglutinin disease		7
mixed-type AIHA		1
Number of patients with lymphoplasmocytic lymphoma		1
Other malignant diseases (N. ovarii)		1
concomitant and prior treatments		
Glucocorticoids		4
Prior Rituximab		1
Laboratory parameters	Local reference level	Median (lower and upper quartile)
Hemoglobin	12-16 g/dL / 13.5-18 g/dL	9.8 g/dL (8.6–10.5 g/dL)
Reticulocyte count	32–110 G/L	110 G/L (73–179 G/L)
Total bilirubin mean	0–1.2 mg/dL	1.5 mg/dL (0.9–1.9 mg/dL)
LDH mean	< 250 U/L	304 U/L (277–377 U/L)
Haptoglobin	30–200 mg/dL	<12 mg/dL
Mean baseline CD20^+^ cell count [per μL]		276 (24–774 /μl)

**Figure 2:**
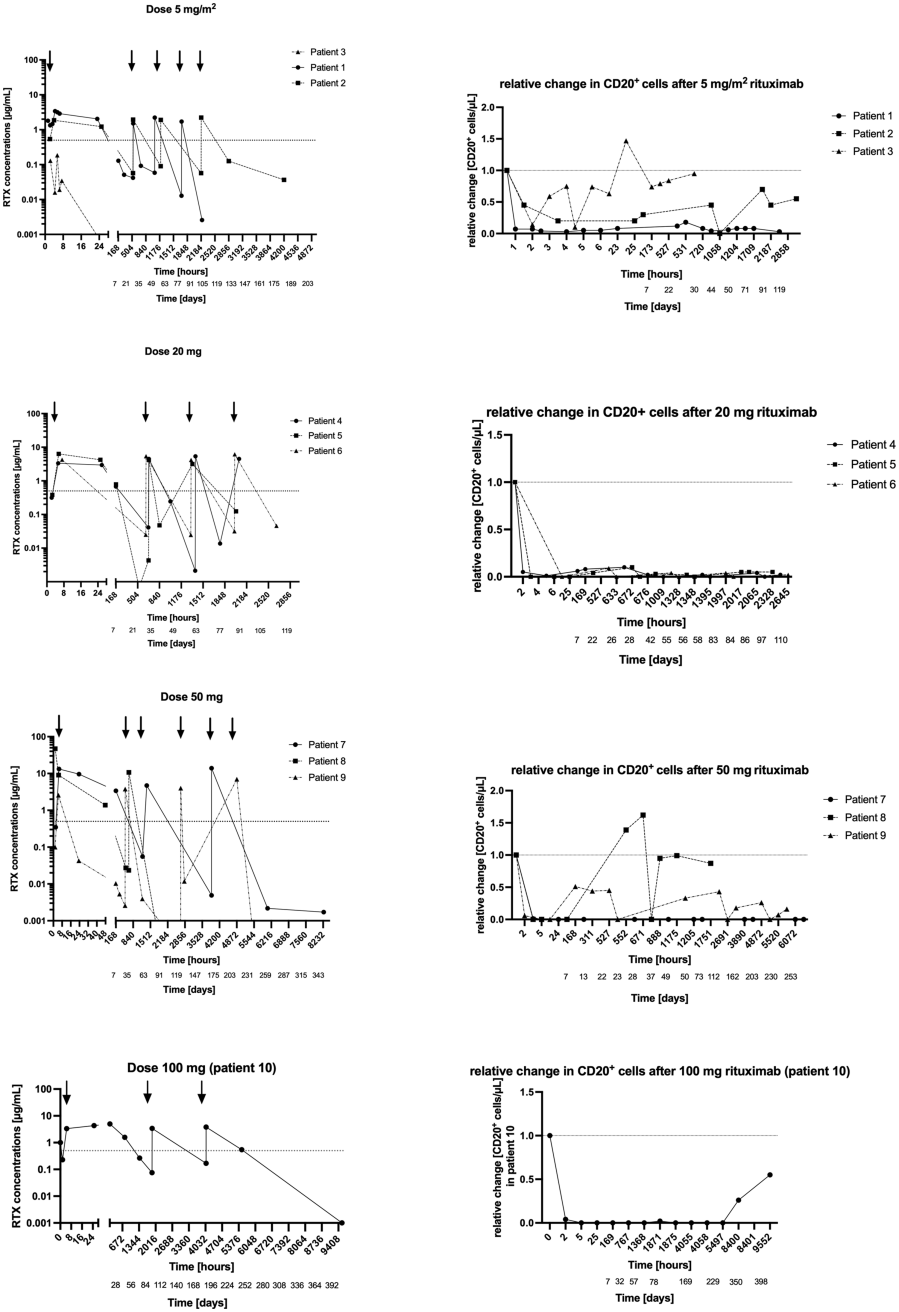
(Left) Rituximab concentration in plasma after 5 mg/m^2^, 20 mg, 50 mg and 100 mg infusions. y-axis = logarithmic scale of absolute rituximab concentration in plasma, x-axis = time in hours and days. (Right) Concomitant CD20^+^ cells. y-axis = relative change in CD20+ cells/μl after rituximab administration, x-axis = time in hours and days.

In the first cohort three patients received 5 mg/m^2^ every 3 weeks, two patients with CAD and one with mixed AIHA. In one patient with CAD the infusion of 5 mg/m^2^ immediately depleted all CD20^+^ cells. In one patient CD20^+^ cells were not successfully depleted (Figure 2). In line with these results, rituximab concentrations remained well below 1 μg/mL at all time-points in this subject.

In the patient with mixed-type AIHA CD20^+^ cells recovered 1 week after the first infusion (14 CD20^+^ cells/μL, 0.1 μg/mL rituximab). However, in this subject CD20^+^ cells were successfully depleted after the second infusion and remained suppressed for the scheduled period. In another patient CD20^+^ cells were successfully depleted for the whole scheduled period.

Based on these results, this dose level was considered partially ineffective, and the dose was escalated.

Three patients received 20 mg fixed dose every 4 weeks (Figure 2), two patients with CAD and one with wAIHA. Rituximab infusion successfully depleted CD20^+^ cells for over 24 h in all three patients.

In one patient with CAD CD20^+^ cells remained successfully depleted over the whole study period (114 days = 3 months and 25 days). In the other patient with CAD CD20^+^ cells recovered 4 weeks after the first infusion (9 CD20+ cells/μL, 0.02 μg/mL rituximab). Yet, after the next infusion, they remained suppressed for the scheduled period. In the patient with wAIHA, CD20^+^ cells re-appeared about 4 weeks after the first infusion (41 CD20^+^ cells/μL, 0.004 μg/mL rituximab), 4 weeks after the third infusion (21 CD20 cells/μL, 0.1 μg/mL rituximab), and at the end of study visit, 2 months after the last infusion (49 CD20^+^ cell/μL, rituximab not measurable).

Therefore, the rituximab dose was escalated to 50 mg every 3 months in the next cohort (Figure 2).

Three patients received 50 mg fixed dose every 3 months (Figure 2), two patients with CAD and one with wAIHA. Rituximab transiently depleted all CD20^+^ cells in these three patients. However, only in the patient with wAIHA CD20^+^ cells remained suppressed for the entire study period (345 days = 11 months and 9 days), while CD20^+^ cells recovered in the other two patients (Figure 2).

In one patient with CAD, CD20^+^ cells recovered 3 weeks after the first infusion (150 CD20^+^ cells/μL, rituximab concentration 0.03 μg/mL), and 1 week after the second infusion (103 CD20^+^ cells/μL, rituximab concentration not measurable). In the other patient with CAD, CD20^+^ cells reappeared every 1–3 weeks (1 week after the first infusion: 270 CD20^+^ cells/μL and rituximab concentration of 0.01 μg/mL, 1 month after the second infusion: 176 CD20^+^ cells/μL, rituximab concentration 0.004 μg/mL, 1 week after the third infusion 96 CD20^+^ cells/μL, rituximab concentration 0.01 μg/mL). Therefore, the rituximab dose was escalated to 100 mg every 3 months.

One patient with CAD received 100 mg fixed dose every 3 months. Rituximab infusion depleted CD20^+^ cells completely after the first infusion and showed sustained depletion over the whole study period. Interestingly, through concentrations of rituximab increased over time. CD20^+^ cells reappeared 4 months after the last infusion (56 CD20^+^ cells/μL, rituximab concentration 0 μg/mL).

We estimated terminal elimination half-lives of rituximab using the last two measurable drug concentrations, which is obviously a limitation. However, estimated half-lives ranged from 51 h (approximately 2 days) to 140 h (approximately 6 days), which is much lower than published half-lives of 21 days (40).

**Figure 3:**
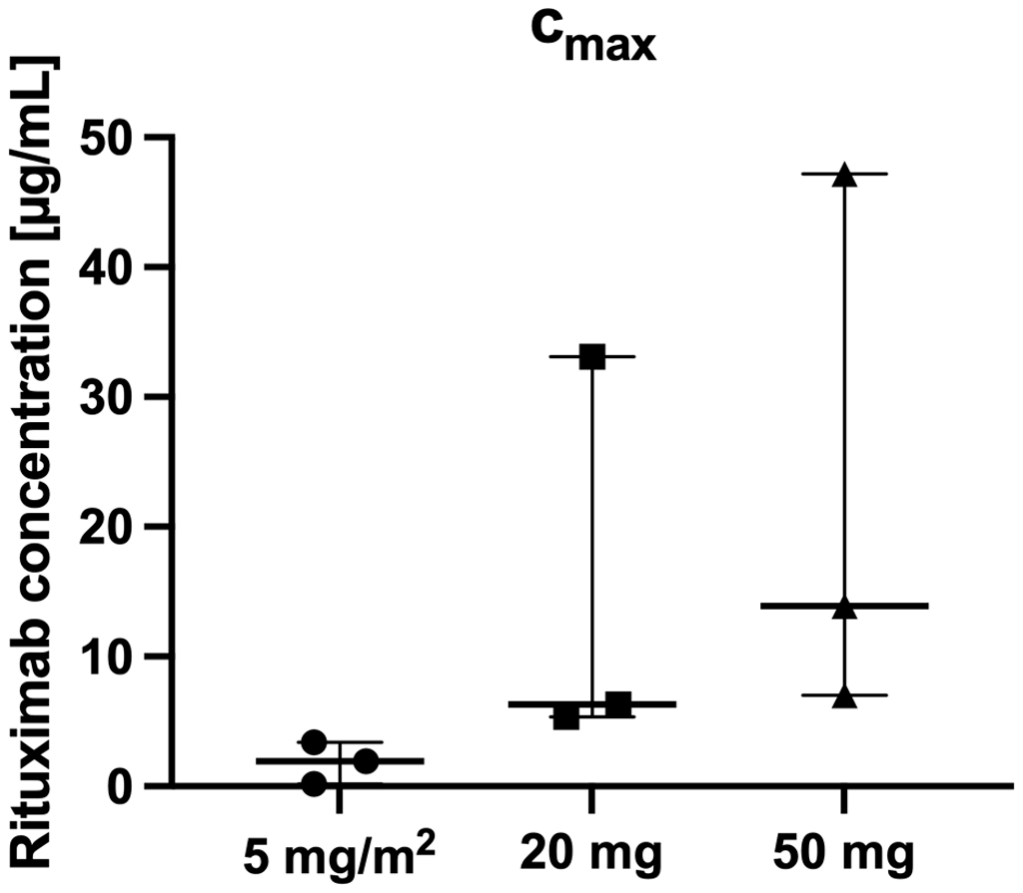
Highest Rituximab c_max_ concentrations per patient over the whole study period (*n* = 3 per group).

**Figure 4:**
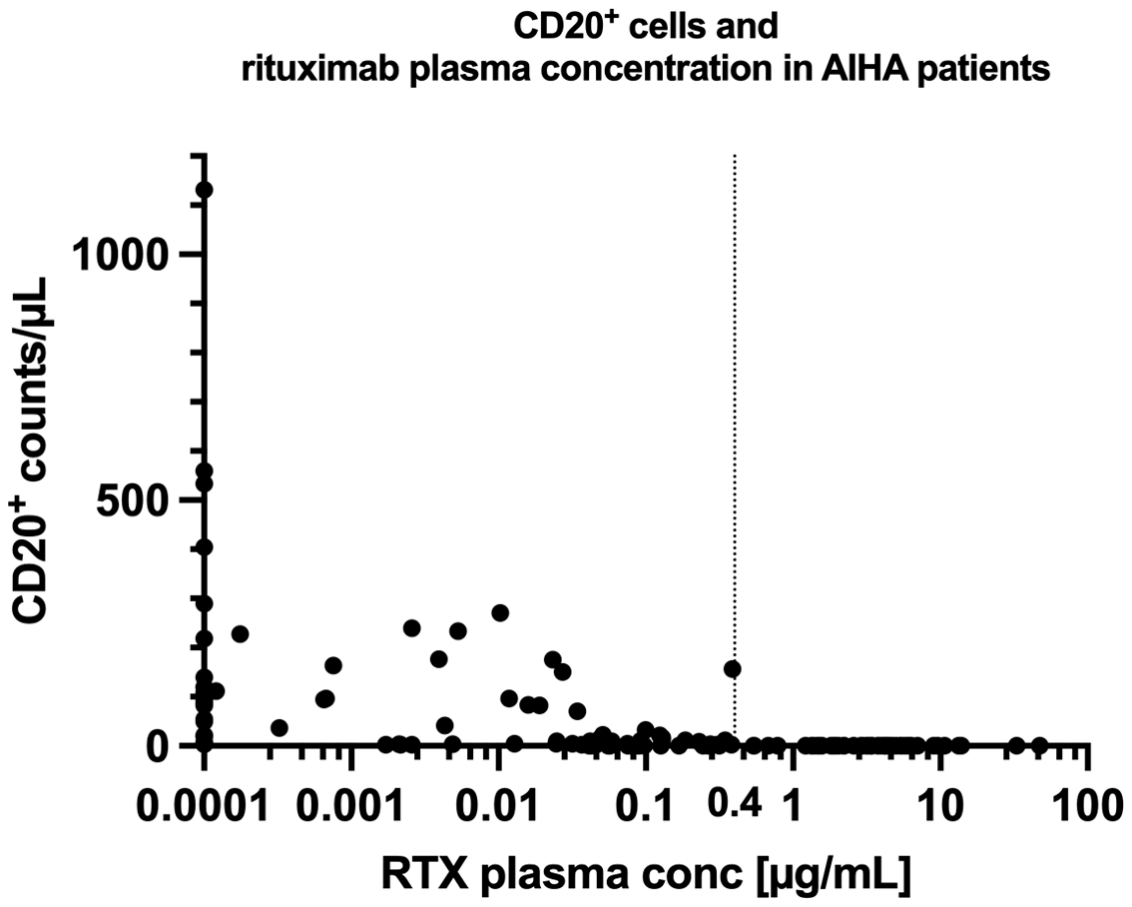
Rituximab concentration and CD20+ cell counts in patients with AIHA. The vertical line at 0.4 μg/mL illustrates the empirically measured EC95% (concentration at which 95% of CD20+ cells are suppressed) of rituximab in our patient cohort. CD20+ cells remained fully suppressed for all concentrations exceeding 0.4 μg/mL. RTX = Rituximab.

There was a close relationship between rituximab plasma concentrations and the number of CD20^+^ cells: CD20^+^ cell counts were below the detection limit if rituximab plasma concentrations were above 0.4 μg/mL in all included patients (Table 2; Figures 3, 4).

**Table 2:** Baseline CD20+ cell counts and Rituximab c_max_ concentrations after first infusion.

	CD 20^+^ cell count per μL at baseline Median (minimum and maximum)	Rituximab concentration c_max_ [μg/mL] after first infusion Median (minimum and maximum)
5 mg/m^2^	111 (20, 119)	1.86 (0.19, 3.41)
20 mg	156 (96, 404)	4.25 (3.33, 6.32)
50 mg	533 (108, 1,131)	9.05 (2.58, 13.30)

### 3.1 Laboratory parameters and emergency treatment

**Figure 5:**
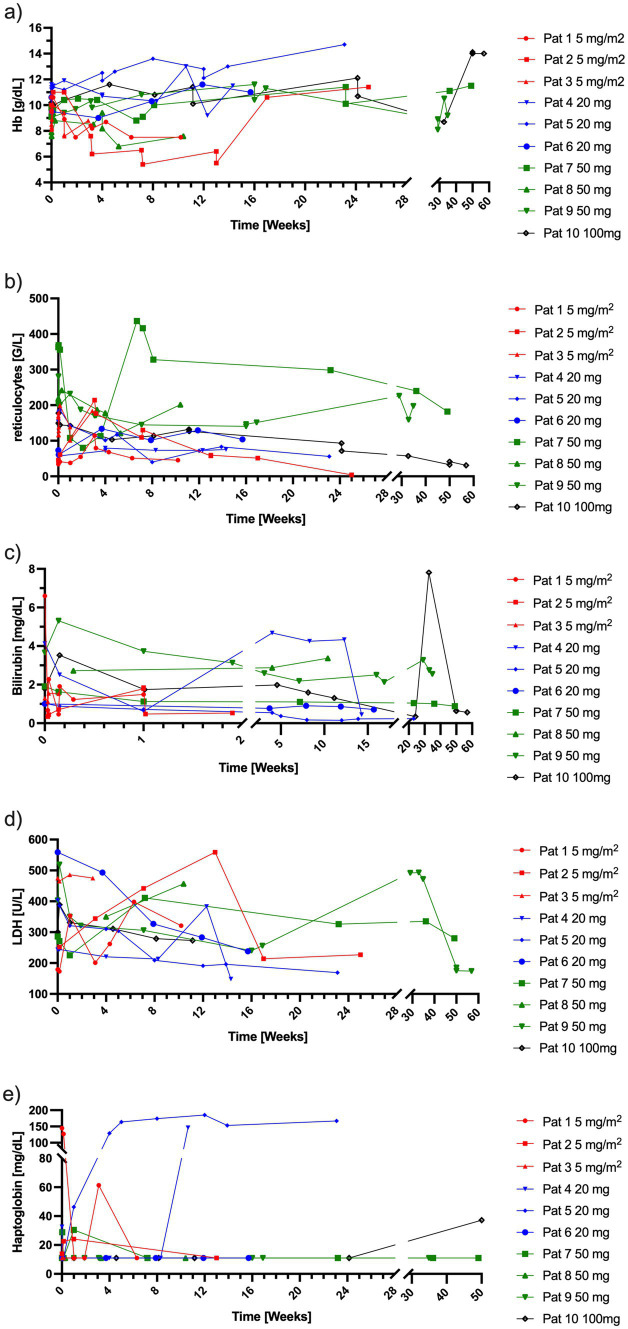
Laboratory parameters indicative of hemolysis per patient over time. (a) Hb concentrations, (b) reticulocyte counts, (c) bilirubin concentrations, (d) LDH concentrations, (e) haptoglobin concentrations. Red = 5 mg/m^2^ of rituximab, blue = 20 mg of rituximab, green = 50 mg of rituximab, black = 100 mg of rituximab. Hb, hemoglobin; LDH, lactate dehydrogenase.

All patients had been pretreated before this trial. Six patients showed signs of ongoing hemolysis with elevated LDH, bilirubin and decreased haptoglobin concentrations. During the study, one patient who received 5 mg/m^2^ needed transfusion of packed red blood cells on two occasions (Hb 7.7 g/dL and Hb 7.5 g/dL) (Figure 5), another patient with 5 mg/m^2^ rituximab received emergency treatment with sutimlimab (41) by the treating hematologists because of signs of hemolysis (Hb 6.5 g/dL, reticulocytes 109.5 G/L, LDH 442 U/L, bilirubin 1.84 mg/dL). One patient (50 mg rituximab) needed a transfusion of packed red blood cells at the end of the trial. One patient (100 mg rituximab) developed endocarditis developed endocarditis 228 days after start of rituximab, which resulted in a reduction of hemoglobin to 8.7 g/dL and an increase in bilirubin. Hemolysis stopped completely in two patients after 20 mg of rituximab, as measured by a normalization of haptoglobin values (Figure 5).

Further details on the individual patients’ response can be found in Supplementary material.

### 3.2 Safety

Two serious adverse events occurred during the study. One non-related serious adverse event was reported in a patient, who had a fall, resulting in a laceration bruise. She was hospitalized overnight for observation. One patient suffered from endocarditis shortly after a vigorous dental cleaning procedure performed at a dentist, which was considered as a possibly related serious adverse event and the same patient also had a prolapsed disc during the study. In general, adverse events were evenly distributed and expectable with regards to the included population and the administered treatment.

## 4 Discussion

The aim of this study was to investigate whether very low doses of rituximab suffice to permanently suppress CD20^+^ cells in patients with AIHA. In total, 10 patients with AIHA completed this study, in whom we observed highly variable responses to the low-dose rituximab regimens. Rituximab infusions fully depleted CD20^+^ cells transiently in all except one patient across all dosing groups and types of AIHA. However, in all treatment groups there were patients with permanently suppressed CD20^+^ lymphocytes and patients in whom the anti-CD20^+^ therapy was ineffective. To optimize recruitment of patients with a rare disease, patients with all types of AIHA were eligible and we did not prespecify to include equal numbers of patients with CAD or warm AIHA. The surplus of CAD patients in this study may be somewhat surprising, but resulted due to chance. Taking the small sample size into account, comparisons of the results between the different subtypes are exploratory, descriptive and should be done only with caution. That said, in our study we did not observe any obvious differences between the different subtypes of AIHA. Successful CD20^+^ cell suppression was observed in CAD as well as wAIHA (one CAD patient in the group of 5 mg/m^2^, one CAD patient in the group of 20 mg and one wAIHA patient in the group of 50 mg). The same is true for patients with therapeutic failure. A previous study observed a lower clinical response rate to low dose rituximab in patients with CAD than in patients with wAIHA, probably due to its different pathogenesis and a higher load of CD20^+^ cells in CAD (32). The small sample size in our study precluded any inferences on clinical effects, especially among the different subtypes of AIHA. However, some CAD patients may have become available for rituximab treatment because they had come off a previous trial (42) or its subsequent named patient program (43, 44).

The initial successful depletion of CD20^+^ cells by low rituximab doses may be restricted to peripheral blood and early CD20^+^ cell reconstitution may be a sign of incomplete removal of these cells from various tissue compartments. One may observe a very close relationship between pharmacokinetics and pharmacodynamics in that regard. Estimated half-lives in our study were considerably shorter (approximately 2–6 days) than those observed in studies using authorized doses (approximately 21 days) (40). This observation is most likely due to ongoing target-mediated drug disposition and incomplete elimination of CD20^+^ cells from tissues and consistent with our findings from healthy volunteers (33).

Target-mediated drug disposition refers to the process by which a monoclonal antibody binds to its target, i.e., the CD20^+^ cells in case of rituximab, with high affinity and builds complexes. These monoclonal-antibody-target complexes are subsequently eliminated by the immune system. Consequently, clearance of the antibody may vary depending on the antigen mass and results in non-linear elimination, especially at low doses (18, 45). In patients with malignant diseases, higher rituximab concentrations and longer half-lives are associated with better survival rates, likely because malignant CD20^+^ cells were completely depleted, which reduces target-mediated drug disposition (18). In that context, we observed that in patients in whom CD20^+^ cells recovered after initially successful depletion these recoveries became less pronounced after each consecutive rituximab dose. It is likely that each rituximab dose depletes part of the CD20^+^ cell pool in tissue and that over time lower doses may suffice to completely remove these cells from all tissues.

Another interesting observation was that rituximab plasma-concentrations of >0.4 μg/mL resulted in a complete depletion of CD20^+^ cells from peripheral blood in all patients. This plasma concentration may be interpreted as the empirically measured EC95% of rituximab *in vivo*. These results confirm our initially hypothesized EC95% of >0.6 μg/mL (33) (Supplementary Figure S1) and support that a trough concentration of >1 μg/mL should be sufficient to permanently deplete CD20^+^ lymphocytes.

However, we observed a high variability in CD20+ cell suppression in the different patients, which may be attributed to various reasons (Supplementary discussion). To overcome the observed variability in CD20^+^ cell suppression, a rituximab loading dose that depletes the entire CD20^+^ cell pool reservoir may be necessary. Maintenance doses that serve the purpose of a long-lasting CD20^+^ cell suppression may be much lower, while being equally effective (46). The dosing regimen may then be adapted to the individual needs of a patient, i.e., aligned with hospital visits that may differ between various diseases, different patients and circumstances of local healthcare systems. Therapeutic drug monitoring (or monitoring of CD20^+^ cells) may be useful to ensure effective treatment with the overall aim of plasma concentrations that exceed the EC95% of 0.4 μg/mL or a complete CD20^+^ cell suppression.

Our sample size was too small to draw definite conclusions on clinical efficacy of low-dose rituximab in AIHA. The main aim of this study was to investigate the pharmacokinetics and pharmacodynamics of various low-dose rituximab regimens focusing on CD20^+^ cell suppression and the study was not designed to prove therapeutic efficacy. Moreover, our dosing regimens were only partly effective in suppressing CD20^+^ cells. Nevertheless, previous studies (31, 32) have already shown that lower doses than those approved (36, 47) do work in AIHA. In a phase II trial (31) a fixed dose of 100 mg rituximab was administered once a week along with 1 mg/kg/day prednisolone. Response [primary outcome defined by increase in Hb levels and normalization of hemolytic parameters (31)] was seen in all 14 patients with warm AIHA after 2 months and was sustained for 12 months. In the 9 CAD patients an overall response rate of 56% was achieved after 2 months with 11–33% relapse rates at 6 and 12 months. Moreover, steroids could be stopped in 65% of warm AIHA and 56% of CAD patients. A follow up study (32) further evaluated the sustained response. Relapse free survival in warm AIHA was 90, 100, 100, and 89% at month 6, 12, 24, and 36 respectively, whereas response was slightly lower in CAD (87, 79, 68, and 68% at 6, 12, 24, and 36 months).

The observed rituximab levels in our patient receiving 100 mg rituximab together with the permanent suppression of 3 months in patients with autoimmune hemolytic anemia (AIHA) to effectively su CD20^+^ cells support Barcellini’s findings (31, 32). We extend them by showing that our proposed dosing regimen (100 mg every 3-month) may suffice to maintain a long-lasting CD20^+^ B-lymphocyte depletion. However, this must be confirmed in larger studies.

Such a dosing regimen could offer economical advantages, also given that the smallest available vial size is 100 mg. Barcellini’s data (31, 32) indicate a highly cost-efficient treatment option for resource limited countries in an off-label condition, where nobody is bound to a specific dose. On a further note, the availability of subcutaneous rituximab formulations may further contribute to improving patient comfort by enabling decentralized, long-term drug administration. However, at the moment, only syringes containing 1,400 mg rituximab are available, which currently limits this option.

### 4.1 Study limitations

The main limitation of this study is the small sample size. Conclusions should therefore be drawn with caution and further clinical trials to support the data would be desirable. The study focused on the pharmacokinetics and pharmacodynamics of rituximab at very low doses and the study duration for the individual patient was relatively short, which additionally precluded conclusions on clinical efficacy. Half-lives were approximated using only the last two available drug concentrations. Another limitation of all interpretations of CD20^+^ cell depletion and reconstitution, is the unknown CD20^+^ cell pool in tissue. We included patients with different forms of AIHA. Differences in the pathophysiology between the subtypes introduce heterogeneity, especially as CAD is associated with clonal B-cell disorders. Also, we only included patients with AIHA. Conclusions for other autoimmune diseases should therefore be drawn with caution. The current data do not provide sufficient evidence to treat patients with low-dose rituximab regimens and future clinical trials are necessary to investigate their clinical efficacy.

In conclusion, low doses of rituximab transiently depleted CD20^+^ cells in almost all patients, but the tested low-dose regimens were able to permanently suppress CD20^+^ cells in only a few patients. Additionally, we empirically identified the *in vivo* EC95% to be at 0.4 μg/mL, which may guide future studies using low-doses of rituximab.

## Data Availability

The raw data supporting the conclusions of this article will be made available by the authors, without undue reservation.
