# Supplementary material for: Very low doses of rituximab in autoimmune hemolytic anemia—an open-label, phase II pilot trial
Source: Front Med (Lausanne). 2024 Dec 20;11:1481333. doi: 10.3389/fmed.2024.1481333 (PMC11695359; doi:10.3389/fmed.2024.1481333)
Supplement: Supplementary file 1 [file Data_Sheet_1.PDF]

Supplemental Material

Very low doses of rituximab in autoimmune hemolytic anemia– an open-label, phase II pilot trial

Discussion

Variability of $CD20^+$ cell suppression

Several factors may explain this variability. First of all, the $CD20^+$ lymphocyte pool differs between patients, with regards to previous anti-$CD20^+$ treatments that may have a long-lasting effect on the $CD20^+$ cell pool. In our population, baseline $CD20^+$ cell counts ranged from 20 – 1131/µL (median 138/µl). Additionally, the ratio between peripheral blood $CD20^+$ cells and tissue compartments remains unknown (Carpenter *et al.*, 2009; Cao *et al.*, 2010; Arndt, Jörns and Wedekind, 2018; Häusler *et al.*, 2018). Although $CD20^+$ cells in circulating blood will be depleted immediately, they may still be present in other compartments (i.e. bone marrow, spleen or lymph nodes). For instance, the patient in whom rituximab was inapt to reduce $CD20^+$ cell counts at all timepoints had a significant splenomegaly - conceivably with an enlarged $CD20^+$ cell pool. In contrast, another patient with CAD and a previous treatment of rituximab, had a permanent depletion of $CD20^+$ lymphocytes after receiving 5 mg/m^2 rituximab, because the previous treatment may have resulted in an overall lower $CD20^+$ lymphocyte number. Furthermore, one patient suffered from lymphoplasmocytic lymphoma, which typically is accompanied with an increased number of circulating $CD20^+$ cells and possibly higher dosing requirements (Treon *et al.*, 2001). Shahaf et al. (Shahaf *et al.*, 2016) nicely demonstrated homeostatic feedback mechanisms of peripheral $CD20^+$ cells on the $CD20^+$ cell generation of the bone marrow and showed increased production of $CD20^+$ cells after peripheral $CD20^+$ cell depletion. Cell recovery is likely dependent on the condition of the bone marrow and impacted by other hematologic diseases. However, $CD20^+$ cell reconstitution within 12 months of

rituximab therapy was highly variable also in patients with various autoimmune diseases and differed between diseases (Thiel *et al.*, 2017).

Supplemental References

1. Arndt, T., Jörns, A. and Wedekind, D. (2018) 'Changes in immune cell frequencies in primary and secondary lymphatic organs of LEW.1AR1-iddm rats, a model of human type 1 diabetes compared to other MHC congenic LEW inbred strains', Immunologic Research, 66(4), pp. 462–470. Available at: https://doi.org/10.1007/s12026-018-9015-6.

2. Cao, Y. et al. (2010) 'An optimized assay for the enumeration of antigen-specific memory B cells in different compartments of the human body', Journal of Immunological Methods, 358(1–2), pp. 56–65. Available at: https://doi.org/10.1016/j.jim.2010.03.009.

3. Carpenter, E.L. et al. (2009) 'Collapse of the CD27+ B-cell compartment associated with systemic plasmacytosis in patients with advanced melanoma and other cancers', Clinical Cancer Research: An Official Journal of the American Association for Cancer Research, 15(13), pp. 4277–4287. Available at: https://doi.org/10.1158/1078-0432.CCR-09-0537.

4. Cohen, S. et al. (2016) 'A phase I pharmacokinetics trial comparing PF-05280586 (a potential biosimilar) and rituximab in patients with active rheumatoid arthritis', British Journal of Clinical Pharmacology, 82(1), pp. 129–138. Available at: https://doi.org/10.1111/bcp.12916.

5. Häusler, D. et al. (2018) 'Functional characterization of reappearing B cells after anti-CD20 treatment of CNS autoimmune disease', Proceedings of the National Academy of Sciences of the United States of America, 115(39), pp. 9773–9778. Available at: https://doi.org/10.1073/pnas.1810470115.

6. Iacona, I. et al. (2000) 'Rituximab (IDEC-C2B8): validation of a sensitive enzyme-linked immunoassay applied to a clinical pharmacokinetic study', Therapeutic Drug Monitoring, 22(3), pp. 295–301. Available at: https://doi.org/10.1097/00007691-200006000-00010.

7. Schoergenhofer, C. et al. (2018) 'Single, very low rituximab doses in healthy volunteers - a pilot and a randomized trial: implications for dosing and biosimilarity testing', Scientific Reports, 8(1), p. 124. Available at: https://doi.org/10.1038/s41598-017-17934-6.

8. Shahaf, G. et al. (2016) 'B Cell Development in the Bone Marrow Is Regulated by Homeostatic Feedback Exerted by Mature B Cells', Frontiers in Immunology, 7, p. 77. Available at: https://doi.org/10.3389/fimmu.2016.00077.

9. Thiel, J. et al. (2017) 'B cell repopulation kinetics after rituximab treatment in ANCA-associated vasculitides compared to rheumatoid arthritis, and connective tissue diseases: a longitudinal observational study on 120 patients', Arthritis Research & Therapy, 19(1), p. 101. Available at: https://doi.org/10.1186/s13075-017-1306-0.

10. Treon, S.P. et al. (2001) 'CD20-directed antibody-mediated immunotherapy induces responses and facilitates hematologic recovery in patients with Waldenstrom's macroglobulinemia', Journal of Immunotherapy (Hagerstown, Md.: 1997), 24(3), pp. 272–279.

Supplementary fig. 1 *(a) Rituximab concentration in plasma at 20 mg, 50 mg and 100 mg respectively and calculated dosing intervals to maintain concentrations above the estimated EC95 and EC50. (b) Calculation of plasma rituximab concentrations at different dosing regimens, with adapted data from Cohen et al.* (Cohen *et al.*, 2016) *Iacona et al.* (Iacona *et al.*, 2000) *and extrapolated from data of the respective study* (Schoergenhofer *et al.*, 2018).

(a)

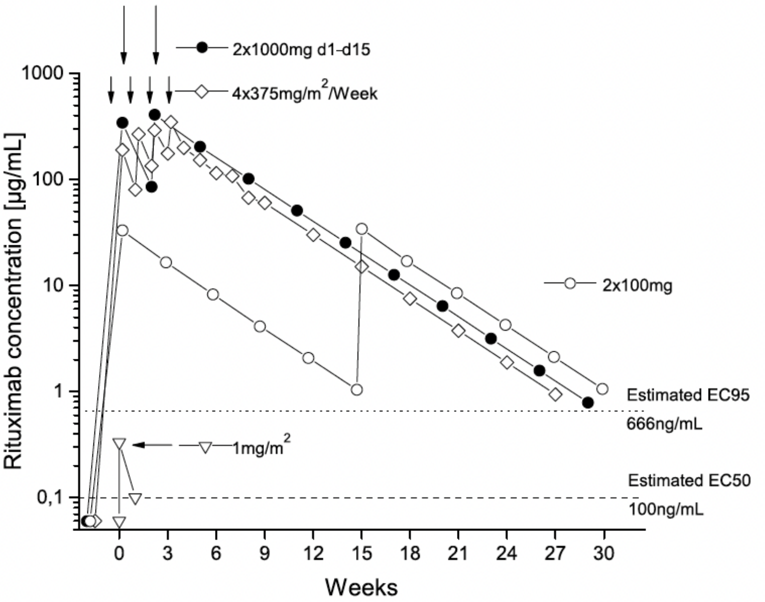

(b)

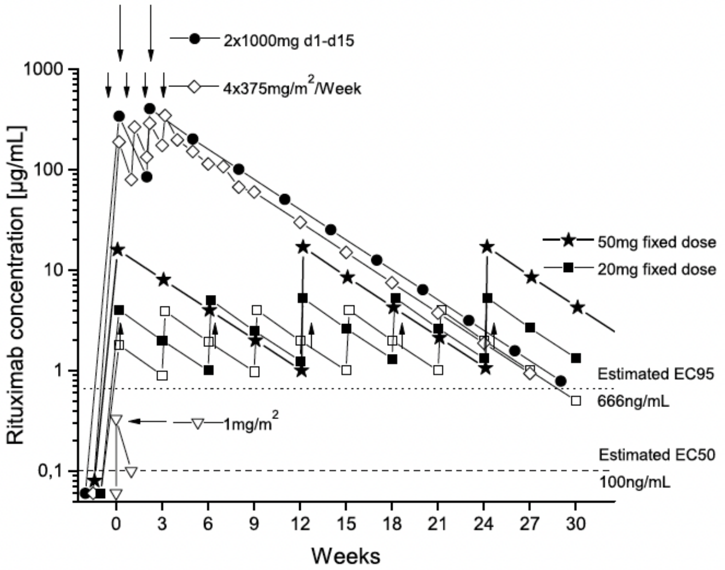

Hemolysis laboratory parameters:

The mean hemoglobin level at baseline was 10.2 ± 0.9 g/dl, the mean reticulocyte count was 145 ± 102 G/L. Mean hemoglobin levels were lowest in patients receiving 5 mg/m^2 and they had greater reductions than those receiving 20 mg and 50 mg, as reflected by the higher standard deviation. Two patients (patient 1 and patient 8) received transfusion of red packed blood cells. Six patients showed signs of ongoing hemolysis with elevated LDH, bilirubin and decreased haptoglobin concentrations.

Short narratives on the clinical course of individual patients:

5mg/m^2 Rituximab

- Patient 1 with CAD had 2 episodes with signs of acute hemolysis (↓ hemoglobin, ↓ haptoglobin, ↑ LDH and ↑ Bilirubin) with the necessity of transfusion of packed red blood cells.
- Patient 2 with CAD had a hemoglobin concentration of 10 g/dL at baseline, which decreased initially to a minimum of 5.4 g/dL. Hemoglobin concentration undulated between 6 -7 g/dL in the early treatment phases. Of note, these low concentrations were partly caused by hemodilution (pre-medication). Hemoglobin concentrations increased to > 10 g/dL in the course of the study, without any further treatment. This development was accompanied by respective improvements in parameters indicative of hemolysis.
- In Patient 3 with mixed-type AIHA the minimum hemoglobin concentration was 7.6 g/dL with signs of ongoing hemolysis. Rituximab concentrations were not measurable and CD20+ cells were not suppressed after infusion, which led to the discontinuation of the study in this subject. (93/µL compared to 111/µL at the beginning of the study)

20 mg Rituximab

- Patient 4 with CAD had hemoglobin concentrations mostly >10 g/dL (nadir 9.2 g/dL), with variable degree of hemolysis according to the parameters of interest (persistent bilirubin elevation, low reticulocytes, undulating LDH inverse to Hb concentrations and normalized haptoglobin at the end of the study)
- Patient 5 with wAIHA had a mild anemia at the beginning of the study and signs of mild hemolysis (↓ haptoglobin, ↑ LDH and ↑ Bilirubin), but hemoglobin concentrations and parameters of hemolysis improved and normalized during the study.
- Patient 6 with CAD had a nadir of 9 g/dL hemoglobin after the first infusion. However, the initially clearly elevated signs of hemolysis improved continuously during the study – which was accompanied by a steady rise in hemoglobin concentration

50 mg Rituximab

- Patient 7 with wAIHA: hemoglobin concentrations undulated between 9 and 10 in the beginning of the study, but hemoglobin improved to approximately 12 g/dL towards the end of the study. Haptoglobin concentrations were continuously reduced and LDH was mildly elevated during the entire observation period, while bilirubin was only mildly elevated at the beginning of the study. Reticulocytes were continuously elevated.
- Patient 8 with CAD had a poor response to rituximab treatment. Hemoglobin values dropped to 7.6 g/dL and the patient required transfusion of packed red blood cells due to ongoing hemolysis (elevated bilirubin and LDH levels and depleted haptoglobin).
- Patient 9 with CAD had mild signs of hemolysis at the beginning of the study, elevated bilirubin and LDH level, but conditions remained stable, hemoglobin concentrations reached a maximum of 11.6 g/dL without the requirement of transfusions. Haptoglobin remained suppressed during the entire study period and all other parameters of hemolysis were slightly elevated during the entire study period

100mg Rituximab

- Patient 10 with CAD had stabile hemoglobin levels throughout the study starting at 10.2 g/dL, mildly elevated LDH and bilirubin at the beginning of the study. Towards the end of the study, patient 10 developed endocarditis, which resulted in a drop in hemoglobin to 8.7 g/dL and an increase in bilirubin concentrations. However, LDH and reticulocytes were not suggestive of acute hemolysis.

In- and Exclusion criteria

Inclusion

- Signed ethic consent obtained before any trial related activities
- Ability to understand the nature and the purpose of the study, including possible risks and side effects; ability to co-operate with the investigator and to comply with the requirements of the trial
- Men or women aged ≥18 years of age with a diagnosis of AIHA in whom treating physicians decide to use rituximab,
- In female subjects either childbearing potential terminated by surgery or one year postmenopausal, or a negative urine pregnancy test during screening and the willingness not to become pregnant during the entire study period by practicing reliable methods of contraception
- Normal findings in medical history and physical examination unless the investigator considers an abnormality to be clinically irrelevant
- Normal laboratory values unless the investigator considers an abnormality to be clinically irrelevant

Exclusion

- Previous treatment with rituximab, or other CD20-antibodies ofatumumab, obinutuzumab or ocrelizumab within 12 months or patients with suppressed B-cell levels
- The recent use of high doses of corticosteroids (>100 mg/day)
- The use of intravenous immunoglobulins, unless cyclic thrombocytopenia occurs
- Planned splenectomy within 3 months or recent splenectomy
- Patients with HepBc antibodies
- Necessity of acute treatment of autoimmune-mediated hemolysis (massive hemolysis)

- Clinically relevant infection (<1 week)
- Relevant history of renal, hepatic, gastrointestinal, cardiovascular, respiratory, skin, hematological, endocrine, inflammatory or neurological diseases, that may interfere with the aim of the study
- Ascertained or presumed hypersensitivity to the active principle and/or formulations' ingredients; history of anaphylaxis to drugs or major allergic reactions in general, which the investigator considers may compromise the safety of the participants
- Use of medication during 2 weeks before the start of the study, which the investigator considers may affect the validity of the study
- Drug abuse, alcohol (>1 drinks/day, defined according to USDA Dietary Guidelines)
- Pregnancy (positive pregnancy test at screening or during study phase), lactation or unreliable contraception in female subjects with child-bearing potential

Females of childbearing potential:

In females of childbearing potential, a pregnancy test was performed at least once per month. All subjects of childbearing potential were informed that performing adequate contraception is necessary, when participating in this trial.
